# Tropical infectious diseases and skin manifestations: a diagnostic framework

**DOI:** 10.1097/QCO.0000000000001183

**Published:** 2026-02-10

**Authors:** Gaia Cologgi, Marianna Giurco, Rebecca Donadoni, Chiara Sepulcri, Matteo Bassetti

**Affiliations:** a University of Genova, Department of Health Sciences; b Infectious Diseases Unit; c Dermatology Unit, IRCCS Ospedale San Martino, Genoa, Italy

**Keywords:** cutaneous manifestation, neglected tropical diseases, tropical infection

## Abstract

**Purpose of review:**

Cutaneous manifestations of tropical and subtropical infections represent a significant clinical and public health concern, affecting both populations in endemic regions and international travelers with significant socioeconomic burden and social stigma. In light of contemporary climate change, increased human mobility and urbanization, these infections have increased in incidence and gained a broader geographical distribution.

Nevertheless, achieving a diagnosis remains challenging due to overlapping clinical presentations, often limited access to advanced diagnostic tools, and a lack of experience among clinicians in nonendemic regions.

**Recent findings:**

This narrative review outlines a pragmatic diagnostic flowchart based on the most common cutaneous manifestations of tropical infections, offering clinicians a practical tool to recognize lesions and to select the most appropriate investigations to support the diagnostic process.

**Summary:**

Tropical cutaneous infections still represent an expanding global challenge, and early recognition is essential to mitigate morbidity. This review seeks to enhance diagnostic confidence and improve clinical management primarily in nonendemic settings.

## INTRODUCTION

Cutaneous manifestations of infectious diseases are a significant challenge in tropical and subtropical regions, causing socio-economic burden in disadvantaged populations, including stigma, isolation, and long-term disability [1^▪^]. Several of these conditions are prioritized by the WHO in the 2021–2030 roadmap for neglected tropical diseases (NTDs), which aims to reduce their global impact. Their importance, however, extends beyond endemic areas, as they represent a leading cause of medical consultations among returning travellers [2,3^▪^].

Diagnosis of cutaneous NTDs remains challenging in endemic areas due to overlapping clinical presentations, limited specialist expertise, and geographical and financial barriers. In this context, telemedicine and mobile health tools [4^▪▪^] may improve access and earlier intervention [5^▪^]. On the other hand, medical personnel from nonendemic areas might be better equipped, but often have limited experience to recognize NTDs [6].

In light of the rapidly changing global landscape – driven by climate change, travels, wars, and urbanization – this review provides a practical approach to assist physicians in the diagnosis of major cutaneous NTDs. 

**Box 1:**
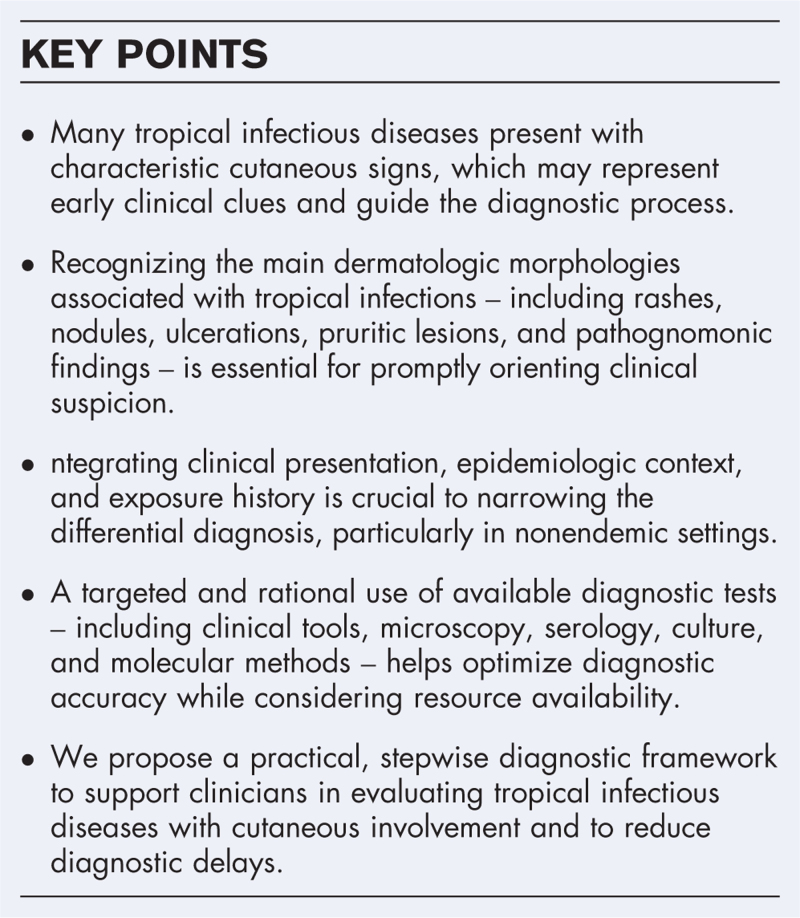
no caption available

## METHODS

For this narrative review, four electronic databases (PubMed, ScienceDirect, Scopus, and Google Scholar) were searched for English-language articles published between 1 January 2024 and 30 September 2025, using free-text terms related to “tropical infection” and “cutaneous manifestation.” Studies without dermatological relevance were excluded. This review does not cover sexually transmitted infections (e.g., syphilis, Mpox, human papilloma virus, HIV-related cutaneous manifestations), globally prevalent conditions (e.g., scabies, tinea capitis, lice, arthropod bites), or noninfectious dermatoses such as sunburns or chemical burns.

The manuscript is structured according to the main dermatological manifestations. For cases with multiple clinical features, only the predominant manifestations were considered. Each pathogen is introduced at first mention only. Visual tools (figures and flow charts) were created using draw. io to support the text.

## GEOGRAPHIC DISTRIBUTION CONSIDERATIONS

**FIGURE 1:**
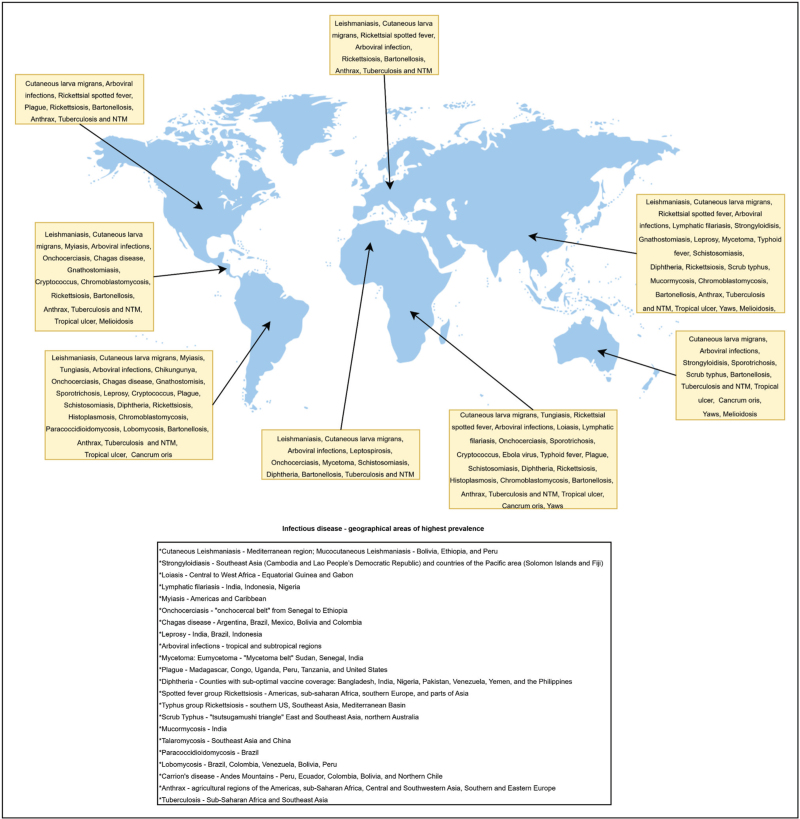
Global map of cutaneous tropical infections. NTM, nontuberculous mycobacteria. All references supporting this figure are cited in the main text.

When a skin and soft tissues infection is suspected, it is essential to collect a thorough medical history to identify essential clues for diagnosis, including travel history (destinations, duration, and timing), lesion morphology, distribution and progression, and associated signs and symptoms [2]. Geographical distribution must also be considered when assessing tropical infectious diseases (Fig. 1). Despite the well described areas of endemicity for the majority of the pathogens discussed in this review, imported cases can be observed virtually all over the world due to globalization and people's migration [6,7], as it occurs with *Gnathostoma* spp. and *T. cruzi*[8,9]. Moreover, the prevalence of infection may differ based on socioeconomic status, as is the case for *Strongyloides* spp. infection [10], or due to poor hygienic conditions [11,12]. Some pathogens, such as *Corynebacterium spp*. can still be relevant in areas where vaccine coverage of the population is insufficient [13]. As previously mentioned, global warming also plays a role in the distribution of infectious diseases: arboviral infections, for example, are constantly expanding worldwide, being endemic in more than 100 countries [14,15].

Moreover, it is important to consider that immunosuppression status, prior medical history can influence disease presentation. Furthermore, it is important to remember that atypical skin presentations are possible [16].

## DERMATOLOGICAL MANIFESTATIONS AND CAUSATIVE PATHOGENS

### Nodules

#### Myasis

Myasis is an ectoparasitosis caused by dipteran fly larvae, that can be classified as “primary”, characterized by larval invasion of intact skin, or as “secondary”, when affecting preexisting cutaneous wounds.

The primary form is associated to the development of gradually enlarging pruriginous papules; additionally, a crawling sensation may be felt. The diagnosis is clinical [17], however, ultrasound may be used to confirm the presence of a larva [18].

#### Onchocercoma

*Onchocerca volvulus* is a filarial nematode responsible for Onchocercarial dermatitis. The classical acute cutaneous manifestation is a pruritic papular eruption; chronic features include papular and lichenified lesions, atrophy, and hyper- or depigmented skin (“leopard skin”). Subcutaneous nodules (*onchocecomas*) are common in endemic areas [19,20].

#### Lobomycosis

After a long incubation period, following a trauma, *L. loboi* typically manifests as painless, ulcerating nodules on the lower limbs [16].

#### Chagas disease

The protozoan parasite *T. cruzi* is transmitted by triatomine bugs; the initial infection is often asymptomatic, when symptomatic, the acute phase (4–8 weeks) is characterized by persistent fever, hepatosplenomegaly, lymphadenopathy, subcutaneous oedema of limbs or face, and morbilliform rash. Inoculation chagomas can also be present. The pathognomic sign is “Romaña's sign”: a unilateral oedema of the palpabrae with conjunctival hyperaemia and preauricular adenopathy.

Acute panniculitis may occur, particularly with latent infection reactivation in transplant patients. If untreated, chronic infection develop (heart and/or gastrointestinal disorders) [21].

#### Leishmaniasis

Leishmaniasis, a zoonosis transmitted by phlebotomine sandflies, includes cutaneous leishmaniasis, mucocutaneous leishmaniasis (MCL), and post–kala-azar dermal leishmaniasis (PKDL) [22^▪^,^23^,^24^].

Cutaneous leishmaniasis begins as a red papule which evolves into a plaque or nodule, or painless circular ulcer with raised borders. Spontaneous resolution is typical, though diffuse cutaneous leishmaniasis with nodular lesions can occur.

MCL is more destructive and typically involves the nose, oral mucosa, and upper respiratory tract. Therefore, a new onset of hearing loss or dysphonia in cutaneous leishmaniasis patients should raise suspicion.

PKDL follows visceral leishmaniasis (*L. donovani)*, and manifests as hypopigmented macular, maculopapular, or nodular lesions around mouth, with possible mucosal involvement [23,25^▪▪^].

#### Bartonellosis: cutaneous bacillary angiomatosis and carrion's disease

Bartonellosis is an arthropod-borne disease that ranges from asymptomatic infection to life-threatening conditions. *B. henselae* (cat-scratch disease) and *B. quintana* (trench fever), are the most prevalent species affecting humans and are both responsible for cutaneous bacillary angiomatosis (cBA), presenting as erythematous to violaceous papules, nodules, or plaques, which may be solitary or multiple [12].

cBA mostly affects skin areas exposed to the vector, and burned skin or preexisting trauma increase the risk of lesion development. *B. bacilliformis* infection causes *Carrion's disease*, characterised by an acute phase of fever and haemolytic anemia. In surviving individuals, clusters of nodular skin lesions (“verruga peruviana”) develop weeks to months later [26,27].

#### Gnathostomiasis

Gnathostomiasis is a food-borne zoonosis caused by the third-stage larvae of *Gnathostoma species*; acquired by ingesting raw or undercooked freshwater fish, amphibians, or reptiles. Cutaneous manifestations include migratory nodular panniculitis, erythematous and edematous swellings, often with pruritus and pain [8].

#### Sporotricosis

*Sporothrix Schenckii* is a dimorphic fungus causing sporotrichosis. Infection results from skin inoculation, but sporadic zoonotic transmission from scratches or animal bites occurs.

Infection commonly involves the skin, subcutaneous tissue, and lymphatic vessels, sometimes spreading systemically. The primary lesion initially develops from a papule or pustule to a subcutaneous nodule, which later ulcerates with secretion of purulent material. Alternatively, the lesion may present as a vegetative, verrucous, plaque-like or tuberous lesion. In about 50% of individuals, a second lesion (sporotrichoid spread) may appear along regional lymphatic vessels [28,29]. Disseminated forms involve multiple noncontiguous lesions, with possible infection of the nasal mucosa or of the conjunctiva [29].

#### Nontuberculous mycobacteria

Nontuberculous mycobacteria (NTM) include pathogens from the Mycobacterium genus except *M. tuberculosis complex* and *M. leprae*. NTM can cause atypical mycobacterial infection, usually resulting in pulmonary disease, but also skin and soft tissues manifestations, presenting with ulcerated or crusted plaques, nodules, and abscesses that develop in a timespan that ranges from a few days to years. *M. marinum* commonly causes nodular lesions at the entry site and one out of three cases develops a sporotrichoid pattern. Less commonly, it can cause abscess, ulcer or pustular lesions [30,31].

#### Leprosy

Leprosy (Hansen's disease), is a chronic infection due to *M. leprae* complex, primarily transmitted via the respiratory route, with an incubation period of two to five years. A potential transmission can follow skin contact with the nine-banded armadillo, a known leprosy reservoir [32].

The main clinical manifestation is a neurocutaneous syndrome with macules, plaques, papules, and nodules (*lepromas)*. These are accompanied by hypopigmentation or hyperpigmentation, reduced sensibility and motor weakness. It can be further classified into “tuberculoid leprosy”, when there are few cutaneous lesions or nerve involvement, strong cell-mediated immunity, and no detectable bacilli, and “lepromatous leprosy”, characterized by diffuse nodular lesions and plaques, no altered sensation, deficient cell-mediated immunity and presence of bacilli [32–35].

#### Cryptococcosis

*Cryptococcus spp* is an environmental yeast, and *C. neoformans* and *C. gattii* are usually responsible for infections in immunocompromised individuals (e.g. HIV/AIDS, transplants recipients, anti-TNF therapy) and occasionally in immunocompetent individuals [36]. Cutaneous and mucocutaneous involvement result from hematogenous spread or, rarely, from direct skin inoculation. Typical skin manifestations are plaque or maculopapular lesions, occasionally ulcerative or infiltrative lesions, usually on exposed areas. Mucosal lesions are less frequent and are generally characterized by multiple ulcers and nodules, with a granulomatous appearance [36–38].

#### Histoplasmosis

Histoplasmosis, or Darling's disease, is an invasive fungal infection caused by *H. capsulatum*, found in soil contaminated with bat or bird faeces. It mainly affects immunocompromised individuals (e.g. CD4 T lymphocyte < 100/mmc, transplants recipient, chemotherapy). The fungus can affect multiple body sites, with the skin involvement typically occurring at areas of previous trauma. Cutaneous involvement often occurs at sites of prior trauma and presents heterogeneously as papules, erythematous plaques, pustules, nodules, or molluscum-like lesions. Early diagnosis is crucial in limiting organ dissemination [39–42].

#### Talaromycosis

*Thalaromyces marneffei* is an invasive mycosis that mainly affects people living with HIV or those with other immunocompromising conditions. It manifests as umbilicated papules in the central portion [16,43].

### Hypopigmented/atrophic lesions

#### Lupus vulgaris

Lupus vulgaris represents the most common form of cutaneous tuberculosis, caused by hematogenous or lymphatic dissemination of *Mycobacterium tuberculosis*.

Cutaneous tuberculosis prevalence is low and may result from direct inoculation, continuous spread or from systemic dissemination. After 2–4 weeks, an ulcerated papular or nodular lesion (tuberculous chancre), develops. The classic lesion is an erythematous plaque with a serpiginous advancing edge and an atrophic and scarring remitting edge [44,45]. Other cutaneous forms include scrofuloderma, orificial tuberculosis, miliary lesions, tuberculous gumma, or tuberculosis verrucosa cutis [45].

### Ulcers

#### Tropical ulcer

A tropical ulcer is typically polymicrobial, often involving *Fusobacterium species*. It presents as a small papule or nodule at site of minor trauma. Subsequently, a malodorous, necrotic and painful ulcer develops [46].

#### Cancrum oris

Cancrum oris or “noma” is a polymicrobial gangrenous infection, often involving *F. necrophorum* and *P. intermedia*. It is characterized by acute necrotizing ulcerative gingivitis with rapid extension of necrosis to the subcutaneous tissues [47].

#### Paraccocidiomycosis

*Paracoccidioides* infection is characterized by ulcers in the upper airways, arms and legs, along with swollen lymph nodes. It is transmitted through inhalation, leading to systemic dissemination of the pathogen. Direct inoculation is a rare occurrence [16].

#### Buruli ulcer

Atypical mycobacteria have already been introduced in Section 1.9; this section focuses on *M. ulcerans*, the etiological agent of Buruli ulcer, a chronic condition that initially presents as a papular, nodular or plaque-like lesion, which evolves to a necrotic ulcer, followed by scarring and spontaneous healing [30,31]. It usually affects individuals exposed to contaminated water sources [48].

#### Cutaneous anthrax

*Bacillus anthracis* is responsible for this zoonotic disease, which primarily infects herbivorous animals, which serve as disease reservoirs. Human infection follows the interaction with infected animals, their products, or carcasses. Intra-human transmission occurs rarely. Cutaneous anthrax (approximately 95% of diagnosed cases), results from the penetration of B. anthracis spores through preexisting skin or mucous lesions. About 1 week after exposure, pruriginous papules develop, which evolve into haemorrhagic vesicles and finally, into a painless ulcer with a central black necrotic eschar [49–51].

#### Yaws

Yaws, caused by *T. pallidum subsp. pertenue*, begins with a painless papule or papilloma that may ulcerate and form a yellow crust. If not treated, secondary yaws develop weeks to years later; it is characterized by disseminated scaly papules and painful hyperkeratotic plaques on palms and soles. Tertiary yaws occur in 10% of untreated cases, causing destructive gummatous lesions of skin, cartilage and bone [52].

#### Melioidosis

Melioidosis, caused by *Burkholderia pseudomallei*, involves the skin in 10–20% of cases. Primary cutaneous lesions include single or multiple abscesses, pustules, cellulitis, or ulcerations. Lesions may be painless or tender and can progress to nonhealing ulcers. Secondary cutaneous involvement from disseminated disease presents as multiple pustules or abscesses [53].

#### Cutaneous diphteriae

The genus Corynebacterium comprises toxin-producing species; *C. diphtheriae* is associated with intra-human transmission, whereas *C. ulcerans* and *C. pseudotuberculosis* are primarily zoonotic infections. Diphtheria is typically marked by potentially life-threatening respiratory symptoms, but skin infections can occur immunocompromised patients. Toxigenic strains form nonhealing ulcers with a thick grey pseudomembrane, whereas nontoxigenic strains produce ulcers with a fibrinous base and erythematous, edematous borders [13]. Abscesses, hidradenitis suppurativa, and trichobacteriosis have also been reported [54].

#### Rickettioses

Rickettsia spp., transmitted by blood-feeding arthropod vectors, are responsible for rickettsioses. After skin inoculation, the pathogen can later disseminate and cause invasive disease. At inoculation site, development of an eschar or “tache noir” is common [55]. Scrub typhus eschars may be atypical, presenting as erythematous, vesicular, erosive/ulcerative, or sclerotic lesions [56].

Depending on the rickettsial group, clinical manifestations may vary:

(1) The spotted fever group of rickettsioses causes febrile illness with macular or maculopapular rash, often involving the palms and soles.
(2) Typhus group is associated with rash and eschar-like lesion at the inoculation site.
(3) The transitional group causes a papulovesicular lesion, which progresses to an eschar with hardened and edematous skin. After 1–2 weeks, systemic symptoms developed togethers with a diffuse cutaneous papular or papulovescicular eruption [55].

#### Mucormycosis

Mucormycosis is a fungal infection caused by *Mucorales*[57], primarily affecting immunocompromised individuals (e.g. with diabetes mellitus, chronic kidney failure, transplant recipients, AIDS), and those with trauma or burns [58]. Infection occurs via inhalation or ingestion of spores, rarely through skin. Cutaneous lesions, usually on the limbs, start as plaques and may progress to gangrene [57,59,60].

### Fever and rash

#### Typhoid fever

*Salmonella typhi* can cause a wide range of infections in humans, including typhoid fever, characterized by fever and systemic spread of the pathogen. In 50% of cases, cutaneous papular lesions (*rose spots),* may manifest. Other reported skin manifestations include abscesses, pustular dermatitis, and, rarely, ulcers, purpura, or petechiae [62–64].

#### Arboviral infections (dengue, zika, chikungunia)

Arboviral infections spread by Aedes mosquitoes include:

(1) *Dengue*: up to 80% of patients develop, 2–7 days after exposure, a maculopapular rash on face, trunk, and limbs that blanches on pressure “islands of white in a sea of red.” Haemorrhagic manifestations may develop, (Herman's rash). During recovery, skin desquamation occurs.
(2) *Zika*: patients typically develop a pruritic maculopapular rash 1–4 days after symptom onset, affecting trunk, limbs, face, palms, and soles, often accompanied by fever, arthralgia, peripheral oedema, and ocular sign.
(3) *Chikungunya:* patients present with a highly pruritic maculopapular rash 3–5 days postexposure; it may persist and evolve into hyperpigmented lesions (“chick sign”/“*brownie nose*”), with vesicular or bullous lesions occurring less commonly [15,65,66].

#### Ebola

It is a viral haemorrhagic fever that causes coagulopathy, haemorrhagic shock, and multiorgan failure. Ebola virus, transmitted through contact with infected body fluids, often produces cutaneous manifestations. After a 2–21 day incubation, a maculopapular rash typically develops [67,68].

### Oedema

#### Mycetoma

Chronic cutaneous mycetoma is a granulomatous infection of subcutaneous tissue. It is possible to distinguish a bacterial form of mycetoma, caused by *Nocardia spp., Actinomadura*, and *Streptomyces*, from a fungal one, associated with *Madurella mycetomatis* and *Falciformispora senegalensis*[69,70].

It typically presents as painless, progressively enlarging subcutaneous lesions with fistulous tracts draining purulent material, most often on the foot after penetrating trauma [71,72].

#### Chromoblastomycosis

*Fonsecaea* and *Cladophialophora spp*. are responsible for chromoblastomycosis, a chronic progressive disease which typically follows trauma; It manifest as a papule, usually on the limbs, which develops into pruritic, verrucous plaques and hyperpigmented areas known as “*cayenne pepper*”, with possible lymphatic dissemination [16,72,73].

#### Lymphatic filariasis

Lymphatic filariasis, also known as elephantiasis in advanced disease, is caused by *W. bancrofti*, *B. malayi*, and *B. timori*. It is characterized by lymphatic dysfunction and chronic lymphedema. From early pitting edema, thickening of skin folds and hyperkeratosis, nodules and verrucous changes develop [20,74].

#### Loiasis

Infection by *Loa loa*, a parasite of the Filaria family, results in Loiasis. The disease vector is a hematophagous horsefly of the Chrysops spp. There are three main cutaneous manifestations:

(1) The eyeworm, a pathognomic sign, results from the passage of adult flariae under the palpebral or bulbar conjunctiva. It might be associated with itching, photophobia, lacrimation, and periorbital oedema.
(2) Skin crawling of the parasite is a visible, palpable, mobile, and pruritic cord, most frequently localized on the forearms.
(3) Calabar swelling is a fleeting and migratory angioedema, firm to palpation and poorly demarcated, possibly erythematous, pruritic, and painful [75].

### Pruritic lesions

#### Cercarial dermatitis

Schistosomiasis results from chronic infection with Schistosoma spp. Larvae, which penetrate skin.

The main manifestation is cercarial dermatitis, a transient urticarial or maculopapular lesion that appears a few hours after skin penetration. To aid the diagnosis of schistosomiasis, C. Iriarte *et al.* suggested a new classification in early and late cutaneous lesions: early cutaneous schistosomiasis, includes dermatitis, urticaria, oedema (Katayama syndrome), and occasionally zosteriform papules developed within 6 months of exposure. Less common lesions are nonpruritic erythematous papules or morbilliform rash.

Late cutaneous disease is rare, usually following visceral involvement, with lesions mainly in perineal or genital areas, ranging from pruritic papules to solitary masses or ulcerated plaques [76–78].

#### Tungiasis

Tungiasis, caused by Tunga penetrans, affects humans and animals. The flea penetrates skin, usually on sand-exposed areas, depositing eggs. Infestation (1–5 days after the inoculation) produces pruritic, erythematous papules that enlarge into nodules (up to 1 cm) and self-resolve as the flea dies 4–6 weeks postinoculation [17,79].

#### Cutaneous larva migrans

Cutaneous larva migrans results from the penetration of nematodes through the epidermis, with *Ancylostoma braziliense* and *Ancylostoma caninum* being the most prevalent. Dogs and cats are natural reservoirs, while humans are accidental hosts. Lesions typically appear as erythematous, slightly raised linear or serpiginous tracks on the feet, often pruritic and varying in length or number [80].

#### Strongyloidiasis

Strongyloidiasis, caused by Strongyloides spp., infects hosts via skin penetration. The pathognomic manifestation of acute strongyloidiasis is a serpiginous urticarial rash (“*larva currens*”). It is a pruritic lesion, which can grow up to 10 cm in length each day, and precedes the development of respiratory and/or gastrointestinal symptoms. Chronic strogyloidiasis can also frequently present with cutaneous symptoms, including *larva currens,* recurrent urticaria and pruritus [10].

## DIAGNOSTIC CONSIDERATIONS

**FIGURE 2:**
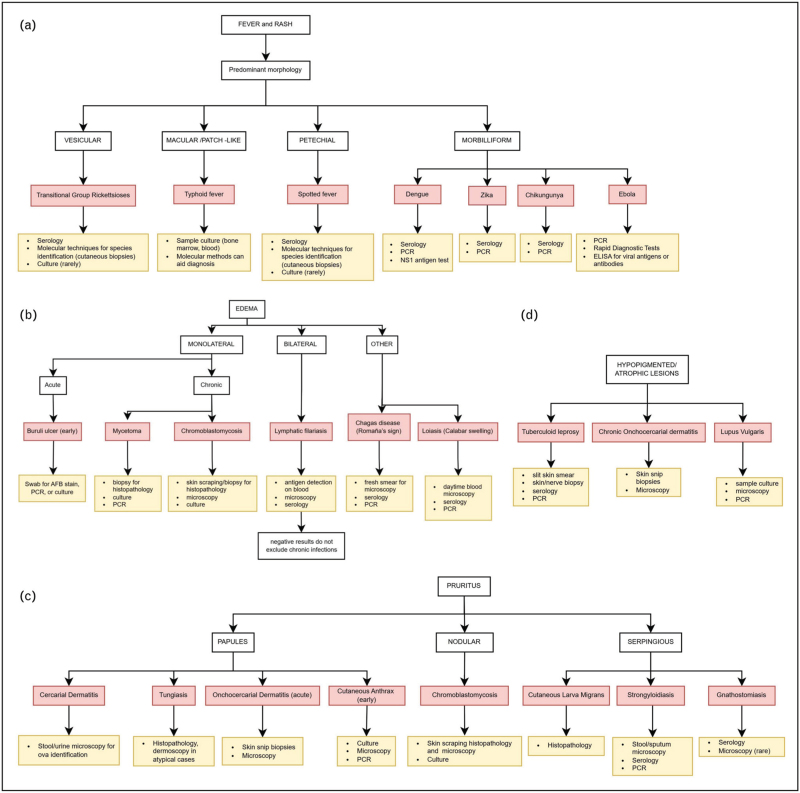
Proposed diagnostic algorithm by clinical presentation. (a) Fever and rash; (b) Oedema; (c) Pruritus; (d) Hypopigmented/atrophic lesions. AFB, acid-fast bacilli; NS1, nonstructural protein 1.

**FIGURE 3:**
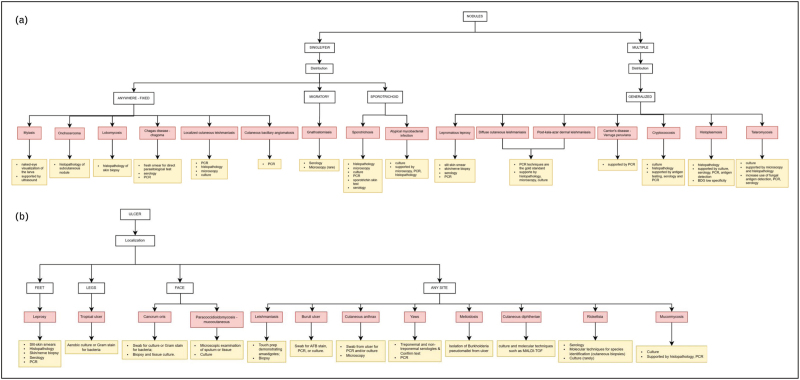
Proposed diagnostic algorithm by clinical presentation. (a) Nodules; (b) Ulcer. AFB, acid-fast bacilli; BDG, beta-D-glucan.

For most pathogens, at least one specific assay is available, as shown in Figs. 2 and 3; however, the choice of targeted testing is guided by clinical suspicion based on presentation, epidemiology, patient history, and resource availability.

Noninvasive stool, urine, or sputum samples may identify schistosome ova or Strongyloides larvae, although these methods require awareness of pathogen tropism, repeated sampling, and expert microscopy [10,77]. Among noninvasive techniques, dermoscopy and ultrasound can further support the diagnosis in atypical or equivocal cases of tungiasis and myiasis, respectively [79,81].

Blood sampling is minimally invasive and broadly applicable to various analyses. Complete blood count detects inflammation, while eosinophilia is common but nonspecific in parasitic infections [8,75]. Serologies can indicate acute or recent infection via seroconversion, antibody titre rise, or IgM detection [15,21,82].

It is important to note that, cross-reactivity, such as between antihelminth antibodies, should be considered [10,75], and serologic tests, such as the Widal Wright one for *S. typhi*, have low sensitivity and specificity, and therefore are not recommended [64]. Negative serology can also occur with infections that are confined to the skin, such as leishmaniasis [83].

Pathogen-specific antigenic testing on blood samples is being investigated for several NTDs, including lymphatic filariasis, dengue, talaromycosis, and cryptococcosis, although for dengue and talaromycosis, antigen detection can also be performed on urine, and for cryptococcosis on cerebrospinal fluid. These tests are gaining popularity but not always widely available [20,36,43,66]. Emerging studies on leishmania are focusing antibodies in urine samples [23,25^▪▪^].

In suspected fungal infections, such as histoplasmosis, serum β-D-glucan exhibits reduced specificity, but it may be considered for skin-limited disease [42].

Direct microscopic evaluation of fresh blood smears might lead to the detection of some parasites, such as microfilariae, with however low sensitivity due to daytime variability [75].

Skin scraping, skin slit biopsies, and traditional cutaneous biopsies support the differential diagnosis of various cutaneous NTDs, with skin scraping useful for chromoblastomycosis for [73], while for most other pathogens, deeper samples are required [32], allowing histopathologic and microscopic assessment, to support the diagnosis in atypical or unclear cases [79,84].

Biologic tissue cultures are often the gold standard for NTDs infections [31,36,64]. However, some pathogens grow slowly [44], others do not grow *in vitro*[35], or require specific media [27,82].

Molecular-based techniques can also support the diagnosis [15,23,44,54,61], but in many cases are expensive and require specialized laboratories [12,23], and for some pathogens lack of standardization, such as *Strongyloides* spp [10].

## CONCLUSION

This narrative review examines the most prevalent cutaneous NTDs, now increasingly relevant beyond endemic regions due to factors such as human mobility and climate change. Early recognition and diagnosis are essential for management and public health control. To assist clinicians, we propose a practical tool, that integrates clinical presentation with a step-by-step diagnostic approach, to guide differential diagnosis and investigations.

## Acknowledgements

*None.*

### Financial support and sponsorship

*This work received no funding.*

### Conflicts of interest

*Outside the submitted work, Matteo Bassetti has received funding for scientific advisory boards, travel, and speaker honoraria from Cidara, Gilead, Menarini, MSD, Mundipharma, Pfizer, Shionogi. The other authors have no conflicts of interest to disclose.*

## REFERENCES AND RECOMMENDED READING

Papers of particular interest, published within the annual period of review, have been highlighted as:

▪ of special interest
▪▪ of outstanding interest

## References

[1▪] Wilder-Smith AB Caumes E. Approach to skin problems in travellers: clinical and epidemiological clues. J Travel Med 2024; 31:taae142. 39485933 10.1093/jtm/taae142

[2] Ending the neglect to attain the Sustainable Development Goals: a road map for neglected tropical diseases 2021–2030. Geneva: World Health Organization; 2020. Licence: CC BY-NC-SA 3.0 IGO.

[3▪] Alderton DL Ackley C Trueba ML. The psychosocial impacts of skin-neglected tropical diseases (SNTDs) as perceived by the affected persons: a systematic review. PLoS Negl Trop Dis 2024; 18:e0012391. 39093848 10.1371/journal.pntd.0012391 PMC11324132

[4▪▪] Gadri S Bounab S Benazi N Zerouak F. A new diagnostic method and tool for cutaneous leishmaniasis based on artificial intelligence techniques. Comput Biol Med 2025; 192:110313. 40359677 10.1016/j.compbiomed.2025.110313

[5▪] Barnowska EJ Fastenau A Penna S. Diagnosing skin neglected tropical diseases with the aid of digital health tools: a scoping review. PLoS Digit Health 2024; 3:e0000629. 39374195 10.1371/journal.pdig.0000629 PMC11458012

[6] Kollipara R Peranteau AJ Nawas ZY. Emerging infectious diseases with cutaneous manifestations: fungal, helminthic, protozoan and ectoparasitic infections. J Am Acad Dermatol 2016; 75:19–30. 27317513 10.1016/j.jaad.2016.04.032

[7] Sousa AS de Vermeij D Ramos AN Luquetti AO. Chagas disease. Lancet 2024; 403:203–218. 38071985 10.1016/S0140-6736(23)01787-7

[8] Liu G-H Sun M-M Elsheikha HM. Human gnathostomiasis: a neglected food-borne zoonosis. Parasit Vectors 2020; 13:616. 33298141 10.1186/s13071-020-04494-4 PMC7724840

[9] de Sousa AS Vermeij D Ramos AN Luquetti AO. Chagas disease. Lancet Lond Engl 2024; 403:203–218. 10.1016/S0140-6736(23)01787-7 38071985

[10] Gordon CA Utzinger J Muhi S. Strongyloidiasis. Nat Rev Dis Primer 2024; 10:6. 10.1038/s41572-023-00490-x 38272922

[11] Akbari M Sheikhi S Akbarzadeh K. Epidemiological insights into human myiasis in Iran: a comprehensive systematic review. Psyche J Entomol 2025; 2025:7475404.

[12] Moulin C Kanitakis J Ranchin B. Cutaneous bacillary angiomatosis in renal transplant recipients: report of three new cases and literature review. Transpl Infect Dis 2012; 14:403–409. 22316326 10.1111/j.1399-3062.2011.00713.x

[13] Hoefer A Seth-Smith H Palma F. *Corynebacterium diphtheriae* outbreak in migrant populations in Europe. N Engl J Med 2025; 392:2334–2345. 40466062 10.1056/NEJMoa2311981

[14] Carbone G Boiardi G Infantino C. Vectors on the move: how climate change fuels the spread of arboviruses in Europe. Microorganisms 2025; 13:2034. 41011366 10.3390/microorganisms13092034 PMC12472643

[15] Sonego B Schettini A Talhari S. Dermatological manifestations during Dengue, Chikungunya, and Zika infections. Curr Opin Infect Dis 2025; 38:92–98. 39773842 10.1097/QCO.0000000000001077

[16] Curtis KL Gold JAW Ritter JM. Dermatologic fungal neglected tropical diseases—Part I. Epidemiology and clinical features. J Am Acad Dermatol 2025; 92:1189–1206. 38852743 10.1016/j.jaad.2024.03.056 PMC11970523

[17] Galván-Casas C Salavastru C Ortiz-Álvarez J. Scabies and other ectoparasitoses. Clin Exp Dermatol 2025; doi: 10.1093/ced/llaf276. 10.1093/ced/llaf276 40567146

[18] Devambez H Richeux M Guericolas M. Eyelid inflammation: an uncommon cause in occidental countries. Am J Emerg Med 2017; 35:1789e3–1789e5. 10.1016/j.ajem.2017.08.021 28888529

[19] Enk CD. Onchocerciasis--river blindness. Clin Dermatol 2006; 24:176–180. 16714198 10.1016/j.clindermatol.2005.11.008

[20] Taylor MJ Hoerauf A Bockarie M. Lymphatic filariasis and onchocerciasis. Lancet 2010; 376:1175–1185. 20739055 10.1016/S0140-6736(10)60586-7

[21] De Sousa AS Vermeij D Ramos AN Luquetti AO. Chagas disease. Lancet 2024; 403:203–218. 38071985 10.1016/S0140-6736(23)01787-7

[22▪] Cosma C Maia C Khan N. Leishmaniasis in humans and animals: a one health approach for surveillance, prevention and control in a changing world. Trop Med Infect Dis 2024; 9:258. 39591264 10.3390/tropicalmed9110258 PMC11598728

[23] Ansari Z Chaurasia A Neha. Comprehensive insights into leishmaniasis: from etiopathogenesis to a novel therapeutic approach. Microb Pathog 2025; 204:107535. 40185168 10.1016/j.micpath.2025.107535

[24] Kato H. Epidemiology of Leishmaniasis: risk factors for its pathology and infection. Parasitol Int 2025; 105:102999. 39592080 10.1016/j.parint.2024.102999

[25▪▪] Villao NV Tabraue-Chavez M Megino-Luque C. A novel colorimetric assay for early differentiation of mucocutaneous and cutaneous leishmaniasis via species-specific identification. Talanta 2025; 293:128016. 40179686 10.1016/j.talanta.2025.128016

[26] Tahmasebi Ashtiani Z Ahmadinezhad M Bagheri Amiri F Esmaeili S. Geographical distribution of Bartonella spp in the countries of the WHO Eastern Mediterranean Region (WHO-EMRO). J Infect Public Health 2024; 17:612–618. 38417187 10.1016/j.jiph.2024.02.009

[27] Rotundo S Tassone MT Marascio N. A systematic review on antibiotic therapy of cutaneous bacillary angiomatosis not related to major immunocompromising conditions: from pathogenesis to treatment. BMC Infect Dis 2024; 24:380. 38589795 10.1186/s12879-024-09253-9 PMC11000314

[28] Yao Q-H Xia X-J Meng X-C. A multivariable model of clinical features for distinguishing sporotrichosis and Mycobacterium marinum cutaneous infection. Diagn Microbiol Infect Dis 2025; 111:116590. 39520774 10.1016/j.diagmicrobio.2024.116590

[29] Barros MBDL De Almeida Paes R Schubach AO. Sporothrix schenckii and Sporotrichosis. Clin Microbiol Rev 2011; 24:633–654. 21976602 10.1128/CMR.00007-11 PMC3194828

[30] Malhotra AM Arias M Backx M. Extrapulmonary nontuberculous mycobacterial infections: a guide for the general physician. Clin Med 2024; 24:100016. 10.1016/j.clinme.2024.100016 PMC11024835 38350409

[31] Mehta N Tyagi M Ramam M Khaitan BK. Cutaneous atypical mycobacterial infections: a brief review. Indian Dermatol Online J 2024; 15:909–919. 39640455 10.4103/idoj.idoj_838_23 PMC11616912

[32] Grijsen ML Nguyen TH Pinheiro RO. Leprosy. Nat Rev Dis Primer 2024; 10:90. 10.1038/s41572-024-00575-1 39609422

[33] Benlamkadam S Errahmany A Raymond K. Hansen's disease: a practical update on a neglected globally significant infection. Cureus 2024; 16:e57374. 38694670 10.7759/cureus.57374 PMC11061821

[34] Mezaiko E Rosa Silva L Paiva Prudente T. Prevalence of oral manifestations of leprosy: a systematic review and meta-analysis. Oral Surg Oral Med Oral Pathol Oral Radiol 2024; 137:362–371. 38262774 10.1016/j.oooo.2023.12.787

[35] Dodge T. Leprosy, ancient, and modern: a review for emergency medicine providers. Adv Emerg Nurs J 2025; 47:108–115. 40106781 10.1097/TME.0000000000000563

[36] Jani A Reigler AN Leal SM McCarty TP. Cryptococcosis. Infect Dis Clin North Am 2025; 39:199–219. 39710555 10.1016/j.idc.2024.11.011

[37] De Sena ACVP De Arruda JAA Oliveira SR. Orofacial cryptococcosis: a challenging clinical report and a systematic analysis of the literature. Int J Surg Pathol 2024; 32:165–181. 37143300 10.1177/10668969231169048

[38] Jalkh AP Sousa NSOD Almeida JDRD. Cutaneous cryptococcosis resembling keratoacanthoma. Rev Soc Bras Med Trop 2025; 58:e0300–2024. 40638454 10.1590/0037-8682-0300-2024 PMC12233740

[39] Mustari AP Rao S Keshavamurthy V. Dermoscopic evaluation of cutaneous histoplasmosis. Indian J Dermatol Venereol Leprol 2023; 91:231–234. 10.25259/IJDVL_889_2022 38031707

[40] Antonson M Borucki R Georgesen CJ. Cutaneous findings as a harbinger of disseminated fungal infection. Cureus 2024; 16:e73944. 39703297 10.7759/cureus.73944 PMC11655184

[41] Cortez-Vila JA Figueroa-Basurto CI Lacy-Niebla RM. Disseminated cutaneous histoplasmosis and its recurrence in an apparently immunocompetent patient. Cureus 2024; 16:e60433. 38883060 10.7759/cureus.60433 PMC11179679

[42] Rodríguez Galvis MC Duarte Villalba MC Chacón Jaramillo PA Usta Stavoli J. Primary cutaneous histoplasmosis: a diagnostic and therapeutic challenge. Int J Dermatol 2025; 65:165–167. 40676821 10.1111/ijd.17974

[43] Wang Y Wang X Xi L. Advancements in diagnosing talaromycosis: exploring novel strategies and emerging technologies. J Fungi (Basel) 2025; 11:434. 40558946 10.3390/jof11060434 PMC12194118

[44] Mohammadnabi N Shamseddin J Emadi M. *Mycobacterium tuberculosis*: the mechanism of pathogenicity, immune responses, and diagnostic challenges. J Clin Lab Anal 2024; 38:e25122. 39593272 10.1002/jcla.25122 PMC11632860

[45] Couppoussamy KI Shanmugam S Devanda R Murugan R. Lupus vulgaris: a narrative review. Int J Dermatol 2024; 63:431–437. 38102852 10.1111/ijd.16987

[46] Veraldi S Faraci AG Valentini D Bottini S. Tropical ulcers: the first imported cases and review of the literature. Eur J Dermatol 2021; 31:75–80. 33648916 10.1684/ejd.2021.3968

[47] Enwonwu CO Falkler WA Phillips RS. Noma (cancrum oris). Lancet 2006; 368:147–156. 16829299 10.1016/S0140-6736(06)69004-1

[48] Anthony M. Buruli ulcer transmission: environmental pathways and implications for dermatologic care. Cutis 2024; 114:184–186. 39879370 10.12788/cutis.1145

[49] Pal M Dhanze H Regassa M. An overview of anthrax: a neglected zoonosis of the tropical region. J Bacteriol Mycol Open Access 2024; 12:13–17.

[50] Ashique S Biswas A Mohanto S. Anthrax: a narrative review. New Microbes New Infect 2024; 62:101501. 39497912 10.1016/j.nmni.2024.101501 PMC11532300

[51] Ochai SO Hassim A Dekker EH. Comparing microbiological and molecular diagnostic tools for the surveillance of anthrax. PLoS Negl Trop Dis 2024; 18:e0012122. 39571005 10.1371/journal.pntd.0012122 PMC11620650

[52] Handley BL Tchatchouang S Marks M. Yaws: a review of clinical features, diagnosis and treatment. Clin Exp Dermatol 2025. 10.1093/ced/llaf100 40036381

[53] Schwartzman G, Reddy SA, Berg SH, *et al*. Cutaneous melioidosis: an updated review and primer for the dermatologist. J Am Acad Dermatol 2023; 89:1201–1208. 10.1016/j.jaad.2023.07.1032 37582471

[54] Mitchell BI Markantonis JE. An underestimated pathogen: *Corynebacterium* species. J Clin Microbiol 2025; 63:e0155224. 40833082 10.1128/jcm.01552-24 PMC12506004

[55] Blanton LS. The rickettsioses. Infect Dis Clin North Am 2019; 33:213–229. 30712763 10.1016/j.idc.2018.10.010 PMC6364315

[56] Lee C-S Kim S You H. Classifying Eschar morphologies: enhancing early diagnosis of scrub typhus. J Korean Med Sci 2025; 40:e234. 40985849 10.3346/jkms.2025.40.e234 PMC12453974

[57] Cutaneous mucormycosis: unveiling rare manifestations - PubMed. [date unknown]. 10.4103/jfmpc.jfmpc_1603_24 PMC12296280 40726652

[58] Skiada A Drogari-Apiranthitou M Roilides E. A global analysis of cases of mucormycosis recorded in the European Confederation of Medical Mycology /International Society for Human and Animal Mycology (ECMM /ISHAM) Zygomyco.net Registry from 2009 to 2022. Mycopathologia 2025; 190:53. 40493110 10.1007/s11046-025-00954-6

[59] Panda S Sahu MC Turuk J Pati S. Mucormycosis: a rare disease to notifiable disease. Braz J Microbiol 2024; 55:1065–1081. 38561499 10.1007/s42770-024-01315-z PMC11153412

[60] Cornely OA Alastruey-Izquierdo A Arenz D. Global guideline for the diagnosis and management of mucormycosis: an initiative of the European Confederation of Medical Mycology in cooperation with the Mycoses Study Group Education and Research Consortium. Lancet Infect Dis 2019; 19:e405–e421. 31699664 10.1016/S1473-3099(19)30312-3 PMC8559573

[61] Tang HM, Chen SC-A, Basile K, Halliday CL. Development and evaluation of a Pan-Mucorales Real-time PCR and a multiplex real-time PCR for detection and identification of Rhizopus arrhizus, Rhizopus microsporus, and Mucor spp. in clinical specimens. *J Clin Microbiol* 2025; 63:e01937-24. 10.1128/jcm.01937-24 PMC12153301 40304523

[62] Marzano A Mercogliano M Borghi A. Cutaneous infection caused by *Salmonella typhi*. J Eur Acad Dermatol Venereol 2003; 17:575–577. 12941099 10.1046/j.1468-3083.2003.00797.x

[63] Lambotte O Debord T Castagné C Roué R. Unusual presentation of typhoid fever: cutaneous vasculitis, pancreatitis, and splenic abscess. J Infect 2001; 42:161–162. 11531326 10.1053/jinf.2000.0783

[64] Kuehn R Rahden P Hussain HS. Enteric (typhoid and paratyphoid) fever. Lancet 2025; 406:1283–1294. 40914181 10.1016/S0140-6736(25)01335-2

[65] Pajor MJ Long B Liang SY. Dengue: a focused review for the emergency clinician. Am J Emerg Med 2024; 82:82–87. 38820810 10.1016/j.ajem.2024.05.022 PMC11254539

[66] Paz-Bailey G Adams LE Deen J. Dengue. Lancet 2024; 403:667–682. 38280388 10.1016/S0140-6736(23)02576-X PMC12372472

[67] Umar SK Diggle MA. The Ebola virus – going beyond the bleeding edge. J Med Microbiol 2025. 10.1099/jmm.0.001998 PMC12224072 40607496

[68] Flórez-Álvarez L De Souza EE Botosso VF. Hemorrhagic fever viruses: pathogenesis, therapeutics, and emerging and re-emerging potential. Front Microbiol 2022; 13:1040093. 36386719 10.3389/fmicb.2022.1040093 PMC9640979

[69] Bonifaz A García-Sotelo RS Lumbán-Ramirez F. Update on actinomycetoma treatment: linezolid in the treatment of actinomycetomas due to Nocardia spp and Actinomadura madurae resistant to conventional treatments. Expert Rev Anti Infect Ther 2025; 23:79–89. 39760435 10.1080/14787210.2024.2448723

[70] van de Sande WWJ Fahal AH. An updated list of eumycetoma causative agents and their differences in grain formation and treatment response. Clin Microbiol Rev 2024; 37:e0003423. 38690871 10.1128/cmr.00034-23 PMC11237709

[71] Alhaj AAM Ahmed ES Hassan A Fahal AH. Epidemiological observations and management challenges in extrapedal mycetoma: a three-decade review of 420 cases. PLoS Negl Trop Dis 2024; 18:e0011841. 38728359 10.1371/journal.pntd.0011841 PMC11111073

[72] Li LX Yoon H. Dematiaceous molds. Infect Dis Clin North Am 2025; 39:75–92. 39701900 10.1016/j.idc.2024.11.006 PMC11786988

[73] Tuckwell W Yesudian PD Chandler D. Chromoblastomycosis: a contemporary review of a neglected disease. Clin Exp Dermatol 2025. 10.1093/ced/llaf201 40367212

[74] Dangare M, Fating T. Journey to an improved quality of life: filarial lymphoedema and physiotherapy rehabilitation. *Cureus*; 2023; 15:e48403. 10.7759/cureus.48403 PMC10700669 38074019

[75] Elouardi C Lefort A Deconinck L. Imported loiasis: diagnostic and therapeutic challenges. Infect Dis Now 2025; 55:105053. 40064469 10.1016/j.idnow.2025.105053

[76] Buonfrate D Ferrari TCA Adegnika AA. Human schistosomiasis. The Lancet 2025; 405:658–670. 10.1016/S0140-6736(24)02814-9 39986748

[77] Schistosomiasis: cercarial finding and recognizing of human hosts as a prerequisite of invasion | Clinical Microbiology Reviews. [date unknown]. 10.1128/cmr.00196-24 PMC12424363 40626643

[78] Iriarte C, Marks DH. Cutaneous schistosomiasis: epidemiological and clinical characteristics in returning travelers. Int J Dermatol 2023; 62:376–386. 10.1111/ijd.16389 36096120

[79] Maco V Maco VP Tantalean ME Gotuzzo E. Histopathological features of tungiasis in Peru. Am J Trop Med Hyg 2013; 88:1212–1216. 23478579 10.4269/ajtmh.12-0645 PMC3752826

[80] Veraldi S Genovese G Cerino U Nazzaro G. Follicular larva migrans. Parasitol Int 2024; 100:102872. 38428565 10.1016/j.parint.2024.102872

[81] Typical histologic features of Tunga penetrans in skin biopsies | Archives of Pathology & Laboratory Medicine. [date unknown]. 10.5858/2002-126-0714-THFOTP 12033962

[82] Blanton LS. The rickettsioses: a practical update. Infect Dis Clin North Am 2019; 33:213–229. 30712763 10.1016/j.idc.2018.10.010 PMC6364315

[83] Aronson N Herwaldt BL Libman M. Diagnosis and treatment of leishmaniasis: clinical practice guidelines by the Infectious Diseases Society of America (IDSA) and the American Society of Tropical Medicine and Hygiene (ASTMH). Clin Infect Dis 2016; 63:e202–e264. 27941151 10.1093/cid/ciw670

[84] Smith MD Procop GW. Typical histologic features of Tunga penetrans in skin biopsies. Arch Pathol Lab Med 2002; 126:714–716. 12033962 10.5858/2002-126-0714-THFOTP

